# Myocardial Matrix Metalloproteinase 3 Protein Expression in Ischemic Heart Failure

**DOI:** 10.3390/ijms27041697

**Published:** 2026-02-10

**Authors:** Vitalija Siratavičiūtė, Dalia Pangonytė, Vaiva Lesauskaitė, Lina Utkienė, Lina Jusienė, Jolanta Marcinkevičienė, Milda Kuprytė, Dovydas Gečys, Vaiva Patamsytė, Zita Stanionienė, Reda Radikė

**Affiliations:** 1 Laboratory of Cardiac Pathology, Institute of Cardiology, Lithuanian University of Health Sciences, 50162 Kaunas, Lithuania; vitalija.sirataviciute@lsmu.lt (V.S.); vaiva.lesauskaite@lsmu.lt (V.L.); lina.utkiene@lsmu.lt (L.U.); lina.jusiene@lsmu.lt (L.J.); jolanta.elena.marcinkeviciene@lsmu.lt (J.M.); milda.kupryte@lsmu.lt (M.K.); zita.stanioniene@lsmu.lt (Z.S.); reda.radike@lsmu.lt (R.R.); 2 Laboratory of Molecular Cardiology, Institute of Cardiology, Lithuanian University of Health Sciences, 50162 Kaunas, Lithuania; dovydas.gecys@lsmu.lt (D.G.); vaiva.patamsyte@lsmu.lt (V.P.)

**Keywords:** heart failure, ischemic heart disease, matrix metalloproteinase 3, immunohistochemistry

## Abstract

Matrix metalloproteinase 3 (MMP3), in conjunction with other MMPs, plays a key role in myocardial remodeling by breaking down the extracellular matrix of the heart and the development of heart failure (HF). However, the existing data on MMP3 protein expression in human myocardium remains insufficient. The objective of the present study was to determine the expression of MMP3 protein in left ventricular myocardial cells at varying stages of ischemic HF. To this end, a quantitative and semi-quantitative immunohistochemical analysis was performed on 113 samples of left ventricular myocardium. A non-selective digital myocardial image analysis revealed that the stage A HF group exhibited higher MMP3 protein expression compared to the control group (*p* < 0.001). Subsequent increases in immunostaining intensity of MMP3 were observed in the stage B HF group (*p* < 0.001). In the stage C/D HF group, MMP3 expression reached its highest level (*p* < 0.001). The intensity of immunostaining exhibited comparable tendencies in both cardiomyocytes and non-cardiomyocytes. Increased levels of MMP3 immunostaining in both types of cells were associated with increased left ventricular mass. Subjects who carried the 5A allele and patients with the 5A homozygous genotype exhibited a higher propensity for increased immunostaining levels in both cardiomyocytes and non-cardiomyocytes when compared to those with the 6A/6A and 5A/6A genotype. These changes in MMP3 protein expression were associated with left ventricular myocardial remodeling in the progression of ischemic HF.

## 1. Introduction

Heart failure (HF) represents a rapidly growing public health issue with global implications, affecting an estimated 64 million individuals worldwide. This increase can be attributed to three factors: the aging population, improved survival rates following myocardial infarction, and enhanced treatment outcomes for patients with HF [1]. A substantial body of research has demonstrated a correlation between progressive myocardial remodeling and subsequent deterioration in left ventricular performance, as well as a less favorable clinical course in patients suffering from HF [2,3,4,5].

Matrix metalloproteinase 3 (MMP3), in conjunction with other MMPs, plays a pivotal role in myocardial remodeling by degrading the extracellular matrix of the heart. MMP3 has been shown to cleave various components, including matrix proteins, growth factors, proteases, surface receptors, and adhesion molecules. Furthermore, it has been demonstrated that this enzyme also processes various pro-MMPs, thereby establishing its synthesis and activation as the primary step in MMP-mediated degradation [6,7,8,9,10].

However, the extant literature on the subject is inconclusive, with only a few scientific studies having shown that increased levels of circulating MMP3 are present in patients with HF [11,12,13,14]. In patients with acute myocardial infarction, plasma MMP3 levels demonstrate a negative correlation with left ventricular ejection fraction. Consequently, MMP3 has been proposed as an independent predictor of left ventricular dysfunction and mortality in patients with myocardial infarction [15,16].

In addition, our understanding of MMP3 protein expression in human myocardium remains limited, as heart tissue samples from patients with HF are rarely available. A quantitative analysis employing confocal microscopy and Western blotting revealed that the levels of MMP3 increased during the progression of HF in pressure-overloaded human myocardium [17]. According to Spinale et al., the levels of MMP3 were elevated in left ventricular myocardium from explanted hearts of patients undergoing total orthotopic heart transplantation due to idiopathic dilated cardiomyopathy. However, these levels remained unchanged in patients with ischemic cardiomyopathy compared to the control group [18]. Fibroblasts, cardiomyocytes, and macrophages are the cells that express MMP3 in the heart; however, as HF progresses, the levels of their expression remain unclear [6,7,8,19]. A recently developed method, single-nuclei sequencing, facilitates the study of gene expression at the cellular level, even in frozen tissue. This methodological advancement enhances the feasibility of studying MMP3 expression in the myocardium. However, the findings of these studies indicated the presence of only negligible levels of MMP3 mRNA in various cell types within the human left ventricular myocardium [20,21,22]. The collective analysis of these studies underscores the necessity to elucidate the expression patterns of MMP3 protein in the diverse cellular compositions of the human left ventricular myocardium through the application of immunohistochemistry.

An investigation into the association between the *MMP3* gene promoter -1171 5A/6A (rs3025058 also known as rs35068180) polymorphism and HF revealed that patients with systolic HF exhibited a higher survival rate if they were carriers of the 6A allele [23]. The *MMP3* 5A/5A genotype has also been identified as an independent predictor of cardiac mortality in patients with non-ischemic HF [24]. Left ventricular dysfunction was found to be more pronounced in patients with acute myocardial infarction and the *MMP3* 5A/5A genotype, and an inverse correlation was observed between circulating MMP3 levels and left ventricular function indicators (fractional shortening and ejection fraction) after a six-month follow-up period [25]. A correlation was also identified between *MMP3* genotypes and circulating protein levels in young patients with ST-segment elevation myocardial infarction [26]. A series of experiments involving the use of cell cultures, including fibroblasts and vascular smooth muscle cells, have demonstrated that the 5A allele exhibits increased promoter activity [27,28,29]. However, this finding has yet to be substantiated in human myocardium.

The objective of the present study was to evaluate the expression of MMP3 protein in left ventricular myocardial cells at different stages of ischemic HF [30]. The present investigation employed quantitative and semi-quantitative immunohistochemical methods, encompassing stages A (at-risk HF), B (pre-HF), and C/D (symptomatic and advanced HF). Furthermore, an evaluation was performed to assess the association between MMP3 protein expression levels and left ventricular mass, which serves as an indicator of myocardial remodeling, as well as the -1171 5A/6A polymorphism of its gene promoter (rs3025058).

## 2. Results

### 2.1. MMP3 Protein Expression

**Figure 1:**
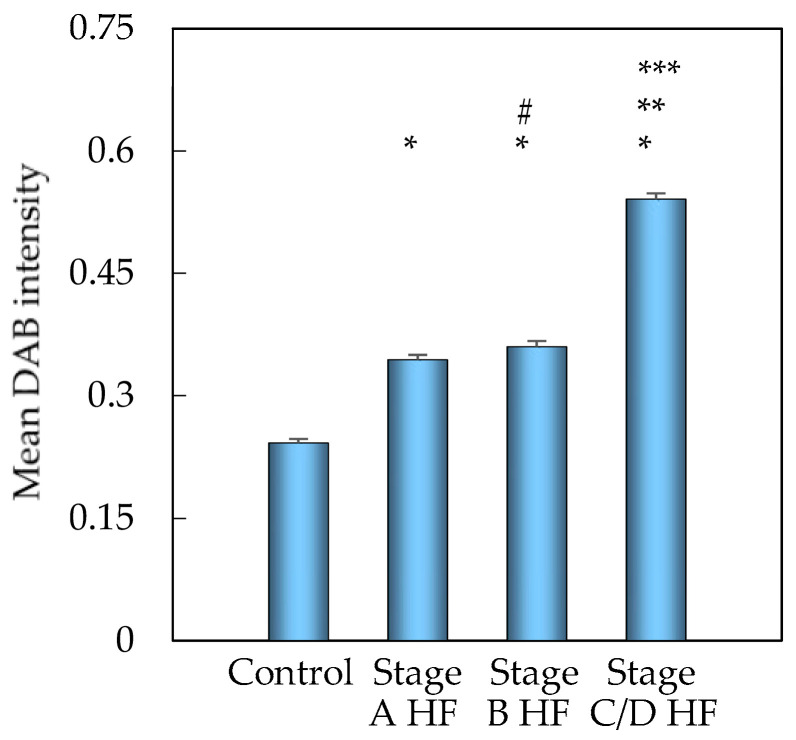
MMP3 protein expression in myocardium of different groups. Bars represent mean and standard error; nested ANOVA with Bonferroni post hoc test was used. * *p* < 0.001—stage A HF, stage B HF, stage C/D HF vs. control group; ** *p* < 0.001—stage C/D HF vs. stage A HF group; *** *p* < 0.001—stage C/D HF vs. stage B HF group; # *p* < 0.01—stage B HF vs. stage A HF group. Abbreviations: HF, heart failure; stages A, B, C, and D HF according to ACC/AHA classification.

A quantitative analysis of digital images revealed a marked increase in myocardial MMP3 immunoreactivity (as measured by DAB-stained optical density) in patients with stage A HF compared to healthy controls (*p* < 0.001, Figure 1). The DAB staining intensity exhibited a further increase in the stage B HF group, surpassing the levels observed in both the control group (*p* < 0.001) and the stage A HF group (*p* < 0.01). The stage C/D HF group demonstrated the most significant expression of MMP3, exhibiting a substantially greater DAB signal compared to the earlier groups (controls, stage A, and stage B HF; all *p* < 0.001).

Furthermore, the intensity of myocardial MMP3 DAB immunostaining displayed a robust positive association with left ventricular mass (Spearman’s rank correlation coefficient r = 0.69, *p* < 0.001).

**Figure 2:**
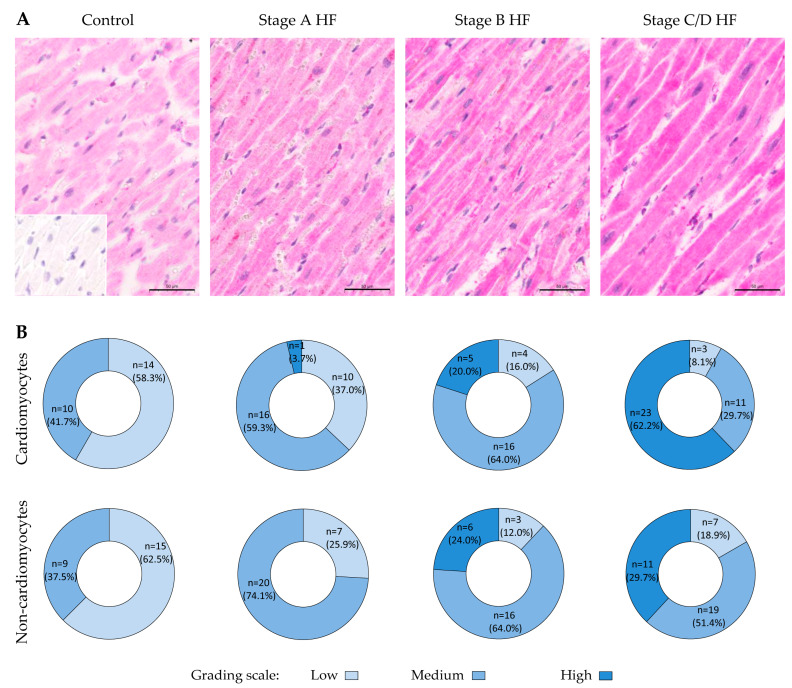
Expression patterns of MMP3 in different groups. (**A**) Representative images of human myocardium immunohistochemistry (antibody ab52915, stained with magenta), which indicates MMP3 deposits in the cytoplasm of cardiomyocytes and non-cardiomyocytes. The staining shows a progressive increase in MMP3 immunopositivity from the control group to stage C/D HF group, with stronger cytoplasmic staining in both types of myocardial cells. The negative control section, which lacks the primary antibody, is in the left lower corner. Scale bar: 50 μm (Images were captured from digital slides, original magnification: 20×). (**B**) Pie charts depict group stratification according to the grading scale. Abbreviations: HF, heart failure; stages A, B, C, and D HF according to ACC/AHA classification. Scale bar: 50 μm.

A semi-quantitative analysis of cardiomyocytes MMP3 immunostaining displayed that the predominant finding in the control group was low cytoplasmic staining of individual cardiomyocytes (in almost 60% of cases), with medium staining in the remaining 40% (Figure 2). In the stage A HF group, medium MMP3 staining was the predominant observation, whereas in the stage B HF group, high MMP3 staining was present in 20% of the cases in addition to the predominant medium immunostaining. In the stage C/D HF group, high MMP3 immunostaining of cardiomyocytes was present in more than 60% of the participants.

Examination of MMP3 immunostaining scores in cardiomyocytes revealed a slight, non-significant increase in the stage A HF group compared to healthy controls. However, the MMP3 score was significantly higher in the stage B HF group than in the control group (*p* < 0.05). There was no significant difference between the stage B and stage A HF groups. The most pronounced elevation occurred in the stage C/D HF group, where the score was higher than in both the control group and the stage A HF group (*p* < 0.001).

Furthermore, there was a strong positive correlation between the score of cardiomyocytes MMP3 immunostaining and the mean myocardial DAB intensity for MMP3 (Spearman’s r = 0.77, *p* < 0.001). Also, there was a moderate yet statistically significant positive correlation between this score and left ventricular mass (Spearman’s r = 0.42, *p* < 0.001).

Semi-quantitative scoring of MMP3 immunoreactivity in non-cardiomyocyte cell populations revealed that the control group predominantly exhibited low-level staining. This pattern was observed in over 60% of individuals (see Figure 2B). Most patients classified as having stage A or B HF exhibited medium MMP3 staining intensity; this pattern was present in nearly 70% of cases. Among stage C/D HF patients, half had medium immunostaining, while one-third had high immunoreactivity.

**Figure 3:**
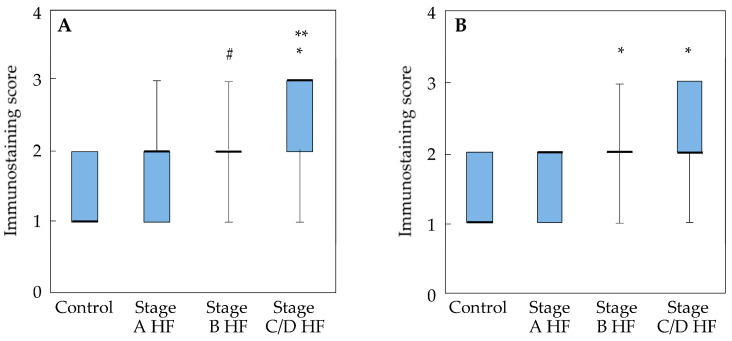
MMP3 protein expression in myocardium of different groups. Semi-quantitative immunohistochemical analysis of cardiomyocytes (**A**) and non-cardiomyocytes (**B**); data are presented as median, the 25th and 75th percentiles, and minimum and maximum values (whiskers); the Kruskal–Wallis test was used in conjunction with Bonferroni-adjusted pair-wise comparisons. # *p* < 0.05—stage B HF vs. control group; * *p* < 0.001—stage B HF, stage C/D HF vs. control group; ** *p* < 0.001—stage C/D HF vs. stage A HF group. Abbreviations: HF, heart failure; stages A, B, C, and D HF according to ACC/AHA classification.

Compared with healthy controls, the non-cardiomyocytes MMP3 immunostaining score in stage A HF patients showed a non-significant upward trend (Figure 3B). In contrast, stage B HF patients exhibited a highly significant increase in MMP3 staining score compared to controls (*p* < 0.001). There was no difference between the stage A and stage B HF groups. In the stage C/D HF cohort, MMP3 immunoreactivity in non-cardiomyocytes was substantially higher than in the control group (*p* < 0.001). However, the immunostaining score in this advanced HF group did not differ significantly from those in the stage A and stage B HF groups.

Furthermore, a significant correlation was observed between the MMP3 immunostaining score in non-cardiomyocytes and the MMP3 immunostaining score in cardiomyocytes (Spearman’s r = 0.59, *p* < 0.001) as well as between the immunostaining score in non-cardiomyocytes and the DAB intensity of myocardial MMP3 (Spearman’s r = 0.60, *p* < 0.001). A significant positive correlation was observed between the non-cardiomyocytes MMP3 immunostaining score and left ventricular mass (Spearman’s r = 0.36, *p* < 0.001).

### 2.2. MMP3 Gene Promoter 5A/6A Polymorphism and Its Association with MMP3 Protein Expression

The genotypic and allelic frequencies of the *MMP3* gene promoter are shown in Table 1. The genotypic distribution of the *MMP3* -1171 5A/6A single nucleotide polymorphism (SNP) (rs3025058) in all groups was consistent with the Hardy–Weinberg equilibrium (*p* > 0.05). The genotypic frequencies of *MMP3* did not differ between the different stages of HF groups, control group and reference group. The frequency of the 5A allele tended to increase with the progression of HF stage, but the difference was not statistically significant (*p* > 0.05).

**Figure 4:**
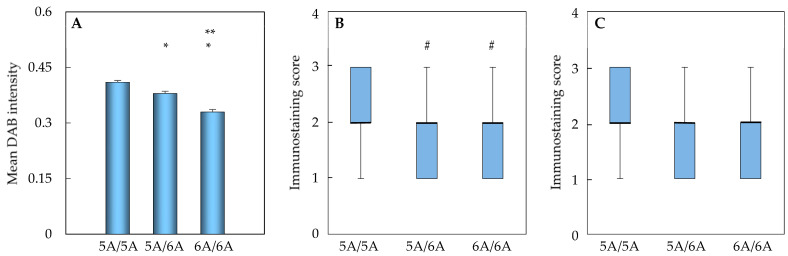
Expression of MMP3 protein according to the SNP of its gene promoter (rs3025058). DAB stain digital image analysis (**A**), bars represent mean and standard error; nested ANOVA with Bonferroni post hoc test were applied. Semi-quantitative immunohistochemical analysis of cardiomyocytes (**B**) and non-cardiomyocytes (**C**); data are presented as median, the 25th and 75th percentiles, and minimum and maximum values (whiskers); the Kruskal–Wallis test with Bonferroni-adjusted pairwise comparisons was used. * *p* < 0.001—5A/6A, 6A/6A vs. 5A/5A subgroup; ** *p* < 0.001—6A/6A vs. 5A/5A subgroup data; # *p* < 0.05—5A/6A, 6A/6A vs. 5A/5A subgroup data.

The highest intensity of MMP3 DAB immunostaining was observed in the myocardium of 5A homozygous patients (Figure 4A). Intermediate intensity of MMP3 immunostaining was observed in heterozygous 5A/6A carriers (*p* < 0.001). The lowest intensity of MMP3 expression was detected in subjects homozygous for the 6A allele. This intensity was statistically significantly different from both 5A homozygotes and 5A/6A heterozygotes (*p* < 0.001). This finding serves to substantiate the established correlation between *MMP3* promoter polymorphism and myocardial MMP3 protein levels.

Stepwise multiple linear regression analysis was performed to identify independent predictors of myocardial MMP-3 protein expression (assessed by DAB intensity). No evidence of multicollinearity was observed among the included variables (all variance inflation factor (VIF) values were close to 1). The final model, comprising left ventricular mass and the 6A allele, accounted for 40.3% of the variability in myocardial MMP-3 protein expression (adjusted R^2^ = 0.403) (Table 2). The unstandardized coefficient (B) for the 6A allele was –0.048 (*p* = 0.041), indicating that the presence of the 6A allele was independently associated with lower MMP-3 protein expression in the myocardium, after adjustment for left ventricular mass.

The MMP3 immunostaining score in cardiomyocytes was found to be highest in patients with the 5A homozygous genotype and was significantly different from the scores in patients with the 5A/6A heterozygous and 6A homozygous genotypes (*p* < 0.001; Figure 4B). A comparable tendency was observed in the MMP3 immunostaining score in non-cardiomyocytes, with higher scores recorded in 5A homozygotes. However, this difference did not reach statistical significance when compared to 5A/6A heterozygotes and 6A homozygotes (*p* > 0.05, Figure 4C).

Univariate logistic regression analysis revealed that higher MMP-3 immunostaining scores in both cardiomyocytes and non-cardiomyocytes were significantly associated with increased left ventricular mass (Table 3). Furthermore, carrying the 5A allele (defined as having at least one 5A allele) was associated with a significantly higher risk of elevated MMP-3 protein expression in both cell types.

Analysis by genotype showed that individuals homozygous for the 5A allele (5A/5A) had a significantly higher likelihood of having high MMP-3 immunostaining than carriers of at least one 6A allele (5A/5A vs. 6A/6A + 5A/6A) in both cell types. However, other genotype comparisons did not reach statistical significance.

Multivariate logistic regression analysis confirmed and expanded upon the results of the univariate analysis (Table 4). VIF values were close to 1 in all models, indicating negligible multicollinearity. Left ventricular mass remained a strong, independent predictor of high MMP-3 immunostaining scores in both cardiomyocytes and non-cardiomyocytes (all *p* ≤ 0.001 across models).

Model 1 revealed that the number of 5A alleles was significantly associated with elevated MMP-3 expression in non-cardiomyocytes (*p* < 0.05), whereas the association in cardiomyocytes was borderline significant (*p* = 0.054). Model 2 revealed that the 5A/5A genotype was significantly associated with higher MMP-3 protein expression in cardiomyocytes compared to the 6A/6A and 5A/6A genotypes (*p* < 0.05). In non-cardiomyocytes, this association approached but did not reach statistical significance (*p* = 0.058).

## 3. Discussion

A multitude of studies have demonstrated the broad spectrum of activity of MMP3 on various components of the extracellular matrix. Nevertheless, the level of MMP3 protein expression in patients with HF remains a relatively understudied area in comparison to the expression of other MMPs.

Our examination aligns with the findings of several studies that have revealed an association between an increased level of MMP3 in plasma and a clinical diagnosis of chronic HF [11,13,14]. Nonetheless, the analysis of patients with chronic HF of ischemic origin has only been conducted in the study of Zile et al. [13]. Furthermore, an increased level of circulating MMP3 protein has been found to negatively correlate with left ventricular ejection fraction in patients with acute myocardial infarction, and has been linked to myocardial remodeling. It has also been proposed as an independent predictor of left ventricular dysfunction and mortality in patients with myocardial infarction [15,16].

However, research on the expression of MMP3 in human myocardium remains limited. According to the findings of a study conducted by Spinale et al., the levels of MMP3 were found to be elevated in the left ventricular myocardium of explanted hearts from patients undergoing total orthotopic heart transplantation due to idiopathic dilated cardiomyopathy. However, the findings indicated that there were no alterations observed in patients diagnosed with ischemic cardiomyopathy when compared with the control group [18]. A quantitative analysis employing confocal microscopy and Western blotting revealed that levels of MMP3 increased during the progression of HF in pressure-overloaded human myocardium [17]. A multitude of experimental studies have demonstrated the presence of MMP3 expression within the myocardium, with the expression observed in various cell types, including fibroblasts, endothelial cells, and cardiomyocytes [6,10,19,31].

Our investigation advances the field by focusing specifically on ischemic HF—a distinct etiology for which data on MMP3 expression has been limited. We provide the first quantitative and semi-quantitative immunohistochemical evaluation of MMP3 protein in human left ventricular myocardium across all ACC/AHA stages of ischemic HF, including early pre-symptomatic stages (A and B). This allows us to map progressive changes in MMP3 expression from at-risk to advanced HF, revealing increases even in stage A. Additionally, we differentiate expression between cardiomyocytes and non-cardiomyocytes. Our immunohistochemical findings detected low levels of MMP3 immunostaining in the cytoplasm of left ventricular cardiomyocytes and non-cardiomyocytes in the control group. In the stage A HF group, increased MMP3 expression was observed in both types of myocardial cell. Subsequent increases in both cardiomyocytes and non-cardiomyocytes in the intensity of MMP3 immunostaining were observed in the stage B HF group. In the stage C/D HF group, the highest levels of MMP3 protein expression were observed in both types of myocardial cells. The finding that MMP3 expression was associated with left ventricular mass suggests that these changes in MMP3 expression are linked to the process of left ventricular remodeling, during which the degradation and synthesis of various extracellular matrix components occur [32,33,34,35,36].

However, single-nucleus sequencing studies have detected very low levels of MMP3 mRNA in various cells of the human left ventricular myocardium [20,21,22]. The results of the immunohistochemical detection of MMP3 protein levels may have been influenced by post-transcriptional regulatory abnormalities, including enhanced translation or reduced protein degradation [6,7]. The poor correlation between MMP3 mRNA and protein levels may also be due to the involvement of MMP3 in dynamic remodeling processes, where protein levels are stabilized by binding to components of the extracellular matrix or tissue inhibitors of MMPs. This finding aligns with the conclusions of other studies, which have demonstrated an increase in MMP3 protein levels in myocardial tissues [17,18].

MMP3 is implicated in a broad array of biological processes, engaging in both matrix-dependent and -independent molecular pathways. Although initially characterized as an extracellular matrix-degrading enzyme, MMP3 has since emerged as a versatile regulator of cellular signaling due to its interactions with various substrates and signaling molecules. These include collagens III, IV, V, IX, X and XI, proteoglycans, laminin, fibronectin, link protein, fibrin, entactin, secreted protein acidic and rich in cysteine, tenascin, vitronectin, pro-MMPs 1, 8, 9 and 13, antithrombin III, plasminogen activator inhibitor-2, α1-proteinase inhibitor, α1-antichymotrypsin, α2-macroglobulin, L-selectin, E-cadherin and heparin-binding epithelial growth factor-like growth factor, among others [6,7,8,9,37,38,39]. This dual functionality enables MMP3 to influence a range of processes, including tissue remodelling, cell proliferation, differentiation, and apoptosis. Its participation in multiple signaling cascades underscores the enzyme’s pivotal function in modulating cellular responses to diverse stimuli.

MMP3 activates various signaling pathways, particularly receptor tyrosine kinase (RTK)-related pathways, through its proteolytic activity. This process involves the release of various factors, including epithelial growth factor, insulin-like growth factor, vascular endothelial growth factor, and transforming growth factor-β (TGF-β). MMP3 has been shown to activate latent TGF-β bound to the extracellular matrix by proteolytically removing its latency-associated peptide [6,40,41]. Subsequent to its release, active TGF-β binds to its receptors, thereby initiating the SMAD-dependent signaling cascade, which modulates fibrosis and cell differentiation [42,43]. Furthermore, MMP3 has been shown to regulate the RTK pathway by affecting the binding of RTK ligands to extracellular matrix components [44,45]. For instance, MMP3 has been shown to cleave tenascin-C, an extracellular matrix glycoprotein that modulates cell adhesion and migration, releasing repeats of the epithelial growth factor that activate RTKs. This cleavage event activates downstream signaling cascades, including the MAPK/ERK pathway, which promotes cell proliferation and survival [6,46,47]. The ability of MMP3 to release RTK ligands further underscores its role as a pivotal modulator of signaling pathways that govern cell behavior. Another pathway influenced by MMP3 is the renin-angiotensin system. MMP3 has been found to play a role in regulating angiotensin II-induced myocardial fibrosis by affecting the activity of extracellular matrix components involved in collagen degradation [48]. The progression of myocardial fibrosis is influenced by the balance between extracellular matrix synthesis and degradation, which is affected by MMP3. It also exerts a direct influence on signaling by modulating the availability of ligands and receptors, including Wnt family molecules and their Frizzled receptors [49,50]. In addition to its extracellular functions, MMP3 has been shown to participate in transcriptional regulation via intracellular signaling through its nuclear localization signals [6]. Through these interactions, MMP3 functions as a molecular switch, regulating the balance between different signaling pathways to maintain cellular equilibrium or driving pathological processes when dysregulated.

The second part of our study is devoted to evaluating the association between myocardial MMP3 protein expression and its gene promoter -1171 5A/6A SNP (rs3025058). In vitro data suggest that the *MMP3* gene promoter 5A/6A polymorphism is functionally significant. The 5A allele has been demonstrated to be associated with higher levels of transcriptional activity, while the 6A allele has been linked to lower activity [27,28,29]. Research on DNA-protein interactions has demonstrated that nuclear proteins exhibit a stronger binding affinity for the 6A sequence compared to the 5A sequence. This observation suggests the possibility that the 6A sequence may function as a transcription repressor [27]. The regulation of MMP-3 expression in vivo is a complex process that has not been thoroughly investigated. The most extensively studied inducible forms of MMP3 are cytokines, including interleukin 1, tumor necrosis factor-α, and TGF-β. These cytokines play pivotal roles in tissue remodeling [51].

The 5A/6A polymorphism in the *MMP3* gene promoter has been previously investigated in patients diagnosed with systolic HF. These patients exhibited a higher survival rate if they were carriers of the 6A allele [23]. The *MMP3* 5A/5A genotype has also been identified as an independent predictor of cardiac mortality in patients with non-ischemic HF [24]. In contrast, data from Faria et al. demonstrate that patients who are 6A homozygous exhibit higher mean values of left ventricular end-diastolic volume and left ventricular end-systolic volume, and lower mean values of left ventricular ejection fraction, in comparison to 5A allele carriers [52]. According to data from Bauters et al., left ventricular end-diastolic volume exhibited no significant differences among *MMP3* genotypes [53]. Moreover, left ventricular dysfunction was found to be more pronounced in patients with acute myocardial infarction and the *MMP3* 5A/5A genotype, and the investigators observed an inverse correlation between circulating MMP3 levels and left ventricular function indicators (fractional shortening and ejection fraction) after a six-month follow-up period [25]. A correlation was also identified between *MMP3* genotypes and circulating protein levels in young patients with ST-segment elevation myocardial infarction [26].

The genotypic and allelic frequencies of the *MMP3* gene were found to be uniform across all groups within our study, exhibiting no significant deviations from the reference group, which was defined as the Lithuanian population. The highest intensity of DAB immunostaining for MMP3 was observed in the myocardium of patients with the 5A homozygous genotype. Intermediate levels of MMP3 immunostaining were observed in heterozygous 5A/6A carriers, while the lowest levels of MMP3 expression were detected in subjects who were homozygous for the 6A allele. A similar trend was observed when the expression levels of cardiomyocytes and non-cardiomyocytes were analyzed separately; the immunostaining score in both types of cells was found to be highest in patients with the 5A homozygous genotype. Furthermore, data analysis revealed that increased levels of MMP3 immunostaining in both cardiomyocytes and non-cardiomyocytes were associated with increased left ventricular mass. Moreover, subjects carrying the 5A allele and patients with the 5A homozygous genotype exhibited a higher propensity for increased immunostaining levels in both cardiomyocytes and non-cardiomyocytes when compared to those with the 6A/6A and 5A/6A genotype. The present study lends support to the hypothesis that the promoter activity of the *MMP3* gene may modify the equilibrium between the synthesis and degradation of the myocardium extracellular matrix proteins, and consequently, the left ventricular function.

From a clinical perspective, the correlation between 5A homozygosity and elevated MMP3 expression, along with left ventricular remodeling, suggests a potential application in risk stratification strategies. Genotyping has the potential to identify patients with ischemic heart disease (especially those who have had a myocardial infarction) who are prone to rapid HF progression, thus prompting the implementation of intensified monitoring or early anti-remodeling therapies, such as angiotensin-converting enzyme inhibitors and others. From a therapeutic aspect, 5A carriers may benefit from MMP3-targeted interventions, such as doxycycline or emerging selective inhibitors, to curb excessive matrix degradation and fibrosis [6,7]. The absence of genotype frequency differences across HF stages can be indicative of this polymorphism’s propensity to modify disease severity (via MMP3 levels) rather than HF susceptibility, consistent with its role as a functional promoter variant. Subsequent research in the form of larger studies may elucidate subtle stage-specific effects.

The limitations of this study must be acknowledged. The process of staining archival formalin-fixed and paraffin-embedded tissues through immunofluorescence is challenging due to the presence of autofluorescence, which is associated with the fixation process. Consequently, the evaluation is constrained in its scope, as it can only encompass non-cardiomyocytes in their collective entirety (a heterogeneous population including fibroblasts, endothelial cells, and immune cells), rather than delving into the intricacies of individual cell origin.

Moreover, given the retrospective nature of this study, it was not possible to ascertain the level of MMP3 protein in blood plasma.

Strengthening the findings would have required cross-validation with independent quantitative methods, such as Western blot, ELISA, or mass spectrometry-based proteomics. However, this was not feasible due to the limitations imposed by the characteristics of the archival material. The tissue was only available in limited quantities. Future research should corroborate these results using multi-omic approaches to improve our understanding of MMP3 dynamics in ischemic HF. Additionally, the present morphological immunohistochemical study was designed to evaluate MMP3 protein levels in human myocardium at different stages of ischemic HF, and it was not related to the use of various medications. We hope that future large-scale clinical trials will clarify this.

## 4. Materials and Methods

### 4.1. Study Design and Groups

Samples of myocardial tissue from the human left cardiac ventricular middle segments were selected from the paraffin tissue blocks archive of the Laboratory of Cardiac Pathology (Institute of Cardiology, Lithuanian University of Health Sciences). In accordance with the American College of Cardiology (ACC)/American Heart Association (AHA) HF classification, a subset of myocardial tissue samples was categorized into three distinct groups based on the HF stages [30], taking into account clinical and morphological data (Table 5).

The stage A HF (at risk for HF) group consisted of individuals who were free of any documented or reported HF symptoms throughout their lifetime. The cases were collected during investigations of sudden cardiac death and through the Kaunas ischemic heart disease register [54]. The clinical data were obtained from the available medical records and via a standardized interview with the relatives of the deceased. Cases were excluded from the study if clinical records were insufficient for staging. The subjects were either healthy or had improved or stabilized prior to death. The subjects exhibited a sudden deterioration within a period of six hours following the manifestation of symptoms indicative of ischemic heart disease [55,56]. A comprehensive macroscopic and microscopic cardiac examination performed at autopsy excluded the presence of any healed (old) myocardial infarction scars. The presence of acute ischemic lesions was found to be histologically consistent with an evolution time of six hours or less [57].

The stage B HF (pre-HF) group comprised individuals who did not manifest symptoms of HF but exhibited left ventricular wall motion abnormalities resulting from a prior myocardial infarction (scar). This finding was also identified during a postmortem morphological investigation. The cases were collected in the same manner as in the previous group.

The C/D HF group included patients with symptomatic HF (present or past symptoms) or advanced HF (severe HF symptoms due to ischemic heart disease that interfere with daily life and result in frequent hospitalizations despite attempts to optimize guideline-directed medical therapy). All subjects in this group had undergone orthotopic heart transplantation. The native hearts removed during the procedure were subjected to systematic gross and microscopic pathological evaluation.

Control subjects were selected from individuals whose cause of death was unrelated to cardiovascular pathology. This group included traumatic/external injuries or abrupt non-cardiac acute medical events. The hearts of these subjects were likewise subjected to complete postmortem morphological examination.

A stringent exclusion criterion prohibited the enrollment of individuals with a documented prior diagnosis of disorders recognized to induce cardiac remodeling and, consequently, MMP3 protein expression levels, across all groups included in the investigation. These disorders included long-standing systemic arterial hypertension, valvular abnormalities (congenital or degenerative/acquired), idiopathic cardiomyopathies, diabetes mellitus, and significant chronic respiratory conditions. This finding was also identified during a postmortem morphological investigation. Prior to conducting immunohistochemical investigations, meticulous conventional histological screening was performed on all candidate cases to ensure tissue suitability. Left ventricular myocardium was carefully sampled to target only zones devoid of fresh ischemic injuries or established post-infarction fibrotic replacement.

Furthermore, in the context of the *MMP3* gene promoter 5A/6A polymorphism study, a reference group (*n* = 740) matched by age and sex (male) was evaluated. The construction of the reference group was executed through the implementation of a frequency-matching design. The reference group was recruited from a random sample of the population of Kaunas. This sample was screened within the international HAPIEE (Health, Alcohol and Psychosocial Factors in Eastern Europe) study, the World Health Organization CINDI (Countrywide Integrated Non-communicable Diseases Intervention) program, and the Kaunas Healthy Aging Study [58,59,60,61]. The subjects were selected at random from the population registry of Kaunas.

The study was conducted in accordance with the Declaration of Helsinki and approved by the Kaunas Regional Biomedical Research Ethics Committee (No. BE-2-77, 15 September 2022).

### 4.2. Immunohistochemistry

Formalin-fixed, paraffin-embedded tissue blocks obtained from the left ventricular myocardium were sectioned to a thickness of 3 µm on a Leica RM2235 rotary microtome (Leica Biosystems, Deer Park, IL, USA). The sections were mounted on Menzel SuperFrost Plus adhesive slides (Menzel, Braunschweig, Germany), air-dried overnight at room temperature, and baked in an oven at 50 °C for at least 12 h to ensure adhesion. Deparaffinization was performed by immersing the slides in xylene, followed by rehydration through a graded series of ethanol concentrations, and final rinsing in distilled water. Heat-induced epitope retrieval was conducted using the RHS-1 microwave processor (Milestone Medical, Bergamo, Italy). The tissue sections were immersed in Tris/EDTA retrieval buffer at pH 9.0 (Agilent Technologies Inc., Wood Dale, IL, USA, Cat. No. S236784-2) and heated to 110 °C for eight minutes.

Immunohistochemical staining was carried out in Sequenza slide racks with the corresponding cover plates (Shandon Diagnostics Limited, Runcorn, UK). Endogenous peroxidase activity was quenched by incubating the slides with a peroxidase-blocking reagent (Agilent Technologies Inc., Wood Dale, IL, USA, Cat. No. S202386-2) for 15 min. The slides were then washed in a wash buffer (Agilent Technologies Inc., Wood Dale, IL, USA, Cat. No. S300685-2). To detect MMP3, two independent polyclonal rabbit anti-human antibodies were used according to the principles recommended for enhanced antibody validation [62]. Ab52915 (Abcam, Cambridge, UK; RRID: AB_881243), directed against amino acids 350–477, was used at a 1:50 dilution, and HPA007875 (Sigma-Aldrich, Merck Group, St. Louis, MO, USA; RRID: AB_1079396), directed against amino acids 219–346, was also applied at 1:50 dilution. Both antibodies were diluted in antibody diluent (Agilent Technologies Inc., Wood Dale, IL, USA, Cat. No. S080983-2) and applied to the sections for 60 min at room temperature. Visualization was achieved using the EnVision FLEX system with a rabbit linker followed by either HRP-conjugated DAB (3,3′-diaminobenzidine) or magenta chromogen (Agilent Technologies Inc., Wood Dale, IL, USA, Cat. No. K800221-2, K800921-2, GV92511-2) development, according to the manufacturer’s protocol. Nuclei were counterstained with hematoxylin (Agilent Technologies Inc., Wood Dale, IL, USA, Cat. No. S330930-2). After counterstaining, the sections were dehydrated in an ascending series of ethanol solutions, cleared in xylene, and mounted with a permanent mounting medium.

Antibody specificity was verified using human kidney sections (3 µm thick) as positive control tissue, where both anti-MMP3 antibodies produced expected staining patterns (comparable to literature-reported MMP3 localization in renal structures). Negative controls included substituting the primary antibodies with isotype-matched, non-immune rabbit IgG at equivalent concentrations and dilutions, resulting in an absence of a specific signal. All staining procedures were performed in parallel and adhered to the best practices established for immunohistochemical reproducibility, including those outlined by expert consensus groups on antibody validation and positive control standardization in diagnostic immunohistochemistry [63].

### 4.3. Image Acquisition and Analysis

The digitization of the stained tissue slides was done using a Pannoramic MIDI scanner (3DHistech, Budapest, Hungary) with a Plan-Apochromat 20×/0.8 NA objective and a Hitachi HV-F22CL camera, resulting in a spatial resolution of 0.2325 µm per pixel.

Digital whole-slide images of myocardial tissue, with MMP3 detected via DAB chromogen, were processed in QuPath version 0.5.1 [64]. Subsequent to the import process, each image was assigned a unique identifier to mitigate selection bias. Within each slide, 15 regions of interest (ROI) were defined at random, with each ROI measuring 235,858 µm^2^. These regions were selected for evaluation. The mean DAB intensity (the intensity of DAB staining, measured as the average optical density in the DAB channel, unitless) measurements were conducted to assess MMP3 expression in each of the selected ROI.

Semi-quantitative scoring was performed using SlideViewer software (Version 2.5) (3DHistech, Budapest, Hungary). For samples stained with a magenta chromogen to visualize MMP3, two independent observers manually annotated all relevant tissue areas. Both cardiomyocytes and non-cardiomyocyte cells were graded using a unified 4-point system. Non-cardiomyocytes were evaluated as a pooled heterogeneous group, including fibroblasts, endothelial cells, macrophages, and other interstitial cells, due to the challenges in cell-specific identification via single-marker immunohistochemistry. This system integrated staining intensity with the proportion of positive cells. This approach followed the standardized protocol described in the Human Protein Atlas [65], with the following categories: 0 = not detected (negative staining or weak positivity in <25% of cells), 1 = low (weak staining in ≥25% of cells or moderate staining in <25% of cells), 2 = medium (moderate staining in ≥25% of cells or strong staining in <25% of cells), and 3 = high (strong staining in ≥25% of cells).

Consensus scores were established by reviewing results from all samples. In ambiguous instances, a board-certified pathologist (D.P.) served as an additional independent reviewer. To maintain objectivity, all images were deidentified prior to analysis, and initial quantifications were performed in a blinded manner. Reproducibility was confirmed through the blinded reevaluation of a subset of representative images by a separate investigator.

### 4.4. DNA Extraction and Genotyping

Genomic DNA was manually isolated from formalin-fixed, paraffin-embedded tissue samples using the Cobas DNA Sample Preparation Kit (Roche Diagnostics, Indianapolis, IN, USA) according to the manufacturer’s instructions. Genomic DNA was isolated from the blood of the reference group subjects as previously described [61]. Concentration and purity were assessed using a Nanodrop 1000 spectrophotometer (Thermo Fisher Scientific, Wilmington, DE, USA). Isolated DNA was stored at −20 °C until analysis.

Genotyping of the *MMP3* -1171 5A/6A SNP (rs3025058) was performed using a HT 7900 real-time PCR quantification system (Applied Biosystems, Foster City, CA, USA) according to the manufacturer’s instructions with the forward primer sequence GTGGCCAAATATTTTCCCTGTATTT, reverse primer GGCACCTGGCCTAAAGACATT, 5A probe (FAM) AAGACATGGTTTTTCCCCCCATCAA and 6A probe (VIC) AAGACATGGTTTTTTCCCCCCATCAA (Thermo Fisher Scientific, Paisley, UK).

### 4.5. Statistical Analysis

The normality of the distribution of continuous variables was assessed using the Shapiro–Wilk test. Continuous variables were expressed as either means with standard deviation (or standard error in nested ANOVA) or as medians with interquartile ranges, as deemed appropriate. The differences between the groups were analyzed using a nested analysis of variance (ANOVA) with post hoc Bonferroni tests for multiple comparisons and a Kruskal–Wallis test with pairwise comparisons adjusted according to Bonferroni’s method. Spearman’s rank correlation test was employed to evaluate correlation trends.

The distribution of MMP3 genotypes was subjected to a chi-square (χ^2^) test to ascertain deviations from the Hardy–Weinberg equilibrium. The association between MMP3 genotypes and study groups (different stages of HF, control group, and reference group) was assessed using the chi-square (χ^2^) test.

A stepwise linear regression analysis was conducted to explore relationships between MMP3 protein expression (by DAB intensity) and left ventricular mass, age, *MMP3* genotypes, and alleles. Univariate and multivariable logistic regression analyses were performed to assess associations between high level of MMP3 protein expression (as determined by immunostaining score) and left ventricular mass, age, *MMP3* genotypes, and alleles. The Akaike Information Criterion (AIC) was employed to select the most suitable inheritance model.

Prior to the implementation of multivariable modeling, an assessment of multicollinearity was conducted using the VIF method. The VIF scores were found to be nearly equal to 1.

Statistical significance was determined at a threshold of *p* < 0.05. All analyses were performed using IBM SPSS Statistics (Version 30.0, IBM, Armonk, NY, USA).

## 5. Conclusions

Our immunohistochemical analyses suggest that alterations in MMP3 protein expression begin in stage A HF (at-risk for HF) and progress through stages B (pre-HF) and C/D (symptomatic/advanced HF). Immunostaining intensity increases in a similar manner in cardiomyocytes and non-cardiomyocytes, and there is a correlation with greater left ventricular mass. Individuals who carry the 5A allele, particularly those who are 5A homozygotes, exhibit elevated levels of MMP3 compared to those with the 6A/6A and 5A/6A genotypes. These alterations contribute to left ventricular remodeling in ischemic HF.

## Figures and Tables

**Table 1:** *MMP3* gene promoter 5A/6A (rs3025058) genotype and allele distribution in HF patients, controls, and reference groups.

	Group					
	HF (All Stages)	Stage A HF	Stage B HF	Stage C/D HF	Control	Reference (Population)
Genotype						
5A/5A	25 (29.1%)	5 (18.5%)	7 (29.2%)	13 (37.2%)	4 (16.7%)	176 (23.8%)
5A/6A	46 (53.5%)	18 (66.7%)	12 (50.0%)	16 (45.7%)	13 (54.2%)	386 (52.2%)
6A/6A	15 (17.4%)	4 (14.8%)	5 (20.8%)	6 (17.1%)	7 (29.1%)	178 (24.0%)
Total	86 (100%)	27 (100%)	24 (100%)	35 (100%)	24 (100%)	740 (100%)
Alleles						
5A	96 (55.8%)	28 (51.9%)	26 (54.2%)	42 (60.0%)	21 (43.8%)	738 (49.9%)
6A	76 (44.2%)	26 (48.1%)	22 (45.8%)	28 (40.0%)	27 (56.2%)	742 (50.1%)
Total	172 (100%)	54 (100%)	48 (100%)	70 (100%)	48 (100%)	1480 (100%)
5A frequency	0.558	0.519	0.542	0.600	0.438	0.499
HW eq						
χ^2^	0.613	3.033	0.001	0.079	0.243	1.384
*p*	0.434	0.082	0.973	0.778	0.622	0.239

Patients (one from stage B HF group and two from stage C/D HF group) with insufficient biological material for MMP3 gene promotor polymorphism analysis were excluded from the study. There was no statistically significant difference in the frequency of MMP3 genotypes and alleles between groups (*p* > 0.05). Abbreviations: HF, heart failure; stages A, B, C, and D HF according to ACC/AHA classification; HW eq, Hardy–Weinberg equilibrium.

**Table 2:** Factors associated with myocardial MMP3 protein expression (by DAB intensity), stepwise linear regression analysis.

Variables	1st Step, Adjusted R^2^ = 0.348		2nd Step, Adjusted R^2^ = 0.403	
	Unstandardized Coefficient B	*p*	Unstandardized Coefficient B	*p*
Constant	0.106	0.080	0.146	0.019
Left ventricular mass	0.002	<0.001	0.002	<0.001
Number of 6A alleles			−0.048	0.041

**Table 3:** Factors associated with high MMP3 protein expression (immunostaining score) in cardiomyocytes and non-cardiomyocytes, univariate logistic regression analysis.

Variables	Cardiomyocytes			Non-Cardiomyocytes		
	OR	CI	*p*	OR	CI	*p*
Left ventricular mass	1.022	1.010–1.034	<0.001	1.024	1.010–1.039	<0.001
Age	1.042	0.995–1.093	0.083	1.048	0.988–1.112	0.116
Number of 5A alleles	2.039	1.035–4.015	0.039	2.731	1.160–6.429	0.021
5A/5A vs. 6A/6A + 5A/6A	3.047	1.206–7.700	0.018	3.200	1.094–9.362	0.034
5A/5A + 5A/6A vs. 6A/6A	1.714	0.526–5.585	0.371	4.732	0.592–37.809	0.143
5A/6A vs. 6A/6A + 5A/5A	0.543	0.228–1.294	0.168	0.715	0.253–2.016	0.525

Abbreviations: OR, odds ratio; CI, confidence interval.

**Table 4:** Factors associated with high MMP3 protein expression (immunostaining score) in cardiomyocytes and non-cardiomyocytes, multivariate logistic regression analysis.

Variables	Cardiomyocytes				Non-Cardiomyocytes			
	OR	CI	*p*	AIC	OR	CI	*p*	AIC
Model 1				108.801				80.846
Left ventricular mass	1.023	1.011–1.035	<0.001		1.026	1.010–1.041	0.001	
Number of 5A alleles	2.048	0.987–4.248	0.054		2.775	1.120–6.873	0.027	
Constant	0.004		<0.001		0.001		<0.001	
Model 2				108.229				81.701
Left ventricular mass	1.023	1.010–1.035	<0.001		1.025	1.010–1.040	0.001	
5A/5A vs. 6A/6A + 5A/6A	3.014	1.088–8.354	0.034		3.085	0.963–9.886	0.058	
Constant	0.007		<0.001		0.002		<0.001	

Abbreviations: OR, odds ratio; CI, confidence interval; AIC, Akaike information criterion.

**Table 5:** Characteristics of the study population by groups.

Characteristic	Group			
	Control *n* = 24	Stage A HF *n* = 27	Stage B HF *n* = 25	Stages C/D HF *n* = 37
Age, mean (SD), years	50.3 (8.4)	54.4 (8.8)	54.8 (7.5)	57.8 (7.5)
Sex	Male	Male	Male	Male
Previous clinical symptoms of HF	No	No	No	Yes
Atherosclerotic stenosis ≥ 75% in at least one coronary artery	No	Yes	Yes	Yes
Old myocardial infarction	No	No	Yes	Yes
Left ventricular mass (free wall without subepicardial adipose tissue), mean (SD), g	103.9 (13.1)	135.1 (22.1)	160.4 (27.5)	194.3 (26.6)

Abbreviations: HF, heart failure; stages A, B, C, and D HF according to the ACC/AHA classification; SD, standard deviation.

## Data Availability

The data that support the findings of this study are available from the corresponding author upon request.

## Abbreviations

The following abbreviations are used in this manuscript:

ACC/AHA	American College of Cardiology/American Heart Association
AIC	Akaike information criterion
CI	Confidence interval
DAB	Diaminobenzidine
HF	Heart failure
MAPK-ERK	Mitogen-activated protein kinase-extracellular signal-regulated kinase
MMP3	Matrix metalloproteinase 3
RTK	Receptor tyrosine kinase
OR	Odds ratio
ROI	Regions of interest
SD	Standard deviation
SNP	Single nucleotide polymorphism
TGF-β	Transforming growth factor-β
VIF	Variance inflation factor
